# Protective effect of HINT2 on mitochondrial function via repressing MCU complex activation attenuates cardiac microvascular ischemia–reperfusion injury

**DOI:** 10.1007/s00395-021-00905-4

**Published:** 2021-12-16

**Authors:** Su Li, Jinxiang Chen, Muyin Liu, Yuqiong Chen, Yuan Wu, Qiyu Li, Teng Ma, Jinfeng Gao, Yan Xia, Mengkang Fan, Ao Chen, Danbo Lu, Enyong Su, Fei Xu, Zhangwei Chen, Juying Qian, Junbo Ge

**Affiliations:** 1 Department of Cardiology, Shanghai Institute of Cardiovascular Diseases, Zhongshan Hospital, Fudan University, 180 Fenglin Road, Shanghai, 200032 China; 2 National Clinical Research Center for Interventional Medicine, Shanghai, China; 3 Department of Cardiology, The Affiliated Suzhou Hospital of Nanjing Medical University, Suzhou Municipal Hospital, Gusu School, Nanjing Medical University, Suzhou, China; 4 Department of Infectious Diseases, Zhongshan Hospital, Fudan University, Shanghai, China; 5 Department of Thoracic Surgery, Zhongshan Hospital, Fudan University, Shanghai, China; 6 Department of Cardiology, Affiliated Hospital of Nantong University, Xisi Road 20, Nantong, Jiangsu China

**Keywords:** Cardiac ischemia–reperfusion injury, Cardiac microvascular injury, HINT2, MCU complex, Mitochondrial calcium overload, Mitochondrial fission

## Abstract

Current evidence indicates that coronary microcirculation is a key target for protecting against cardiac ischemia–reperfusion (I/R) injury. Mitochondrial calcium uniporter (MCU) complex activation and mitochondrial calcium ([Ca^2+^]_m_) overload are underlying mechanisms involved in cardiovascular disease. Histidine triad nucleotide-binding 2 (HINT2) has been reported to modulate [Ca^2+^]_m_ via the MCU complex, and our previous work demonstrated that HINT2 improved cardiomyocyte survival and preserved heart function in mice with cardiac ischemia. This study aimed to explore the benefits of HINT2 on cardiac microcirculation in I/R injury with a focus on mitochondria, the MCU complex, and [Ca^2+^]_m_ overload in endothelial cells. The present work demonstrated that HINT2 overexpression significantly reduced the no-reflow area and improved microvascular perfusion in I/R-injured mouse hearts, potentially by promoting endothelial nitric oxide synthase (eNOS) expression and phosphorylation. Microvascular barrier function was compromised by reperfusion injury, but was repaired by HINT2 overexpression via inhibiting VE-Cadherin phosphorylation at Tyr^731^ and enhancing the VE-Cadherin/β-Catenin interaction. In addition, HINT2 overexpression inhibited the inflammatory response by suppressing vascular cell adhesion molecule-1 (VCAM-1) and intercellular adhesion molecule-1 (ICAM-1). Mitochondrial fission occurred in cardiac microvascular endothelial cells (CMECs) subjected to oxygen–glucose deprivation/reoxygenation (OGD/R) injury and resulted in mitochondrial dysfunction and mitochondrion-dependent apoptosis, the effects of which were largely relieved by HINT2 overexpression. Additional experiments confirmed that [Ca^2+^]_m_ overload was an initiating factor for mitochondrial fission and that HINT2 suppressed [Ca^2+^]_m_ overload via modulation of the MCU complex through directly interacting with MCU in CMECs. Regaining [Ca^2+^]_m_ overload by spermine, an MCU agonist, abolished all the protective effects of HINT2 on OGD/R-injured CMECs and I/R-injured cardiac microcirculation. In conclusion, the present report demonstrated that HINT2 overexpression inhibited MCU complex-mitochondrial calcium overload-mitochondrial fission and apoptosis pathway, and thereby attenuated cardiac microvascular ischemia–reperfusion injury.

**Supplementary Information:**

The online version contains supplementary material available at 10.1007/s00395-021-00905-4.

## Introduction

The reperfusion strategy is the most widely acknowledged treatment for acute myocardial infarction (MI) [22]. However, resupply blood with ischemic myocardium causes ischemia–reperfusion (I/R) injury, which is a clinical reality and a problem that needs much more attention [22]. In recent years, the importance of microcirculation in the protection of cardiac function has become more apparent [18, 19]. The mechanisms of I/R injury to coronary microvascular impairment range from microembolization, impaired vasomotion, capillary plugging and rupture, to leukocyte infiltration, which are partly different from those contributing to cardiomyocyte injury [18, 19]. Moreover, these pathological changes further progress to cardiac edema and even coronary no-reflow phenomena, thereby discounting the benefits of reperfusion therapy [18]. Considering that coronary microvascular dysfunction has been recognized as a determinant for patients’ adverse outcomes, it is highly necessary to better understand the underlying mechanisms and search for effective therapeutic targets [8, 22].

Mitochondria are dynamic organelles that perform self-repair via mitochondrial fission and fusion, two distinctly opposite processes in which one separates the damaged fractions from the whole mitochondrial body and the other merges two fractions into a healthy mitochondrion [39]. The balance of fission and fusion is critical to maintain mitochondrial and endothelial function [7, 28]. However, mitochondrial fission in the coronary microcirculation is excessively activated after reperfusion, which further accentuates mitochondrial reactive oxygen species (ROS) accumulation, mitochondrial permeability transition pore (mPTP) opening, and apoptosis activation, ultimately resulting in microvascular dysfunction [51]. Additionally, reperfusion injury-elicited mitochondrial dysfunction has been implicated in ROS bursts, inflammatory response activation, endothelial swelling, leukocyte aggregates, and altered vasomotion, all of which inevitably contribute to microthrombus formation and microvascular obstruction [13]. In contrast, treatments aimed at mitochondrial repair and ROS elimination alleviated microvascular dysfunction under I/R injury and showed the potential to relieve the no-reflow phenomenon [48]. The above evidence strongly supports the targeting of mitochondrial fission and function as a promising approach for cardiac and microvascular protection from I/R injury.

Calcium overload has been identified as an early sign of apoptosis in cardiomyocytes after I/R injury [41]. Persistent calcium oscillation triggers mitochondrial dysfunction, leads to oxidative stress and mPTP opening, and ultimately activates mitochondrion-dependent apoptosis [36]. This death pattern contributes to many types of cardiovascular diseases, including cardiac I/R injury and heart failure [10, 25]. However, the pathological role of calcium overload in microvascular reperfusion injury is a neglected target for cardioprotection. Limited evidence has proven that the restriction of endothelial mitochondrial calcium ([Ca^2+^]_m_) influx exerted benefits on cardiac microcirculation, as evidenced by enhanced microvascular perfusion, reduced microvascular obstruction, inhibited inflammatory cell infiltration, and decreased infarction size in a mouse cardiac I/R model, and the potentially involved mechanisms were largely attributed to mitigated mitochondrial morphological and functional damage [27, 47]. [Ca^2+^]_m_ influx is primarily mediated by the mitochondrial calcium uniporter (MCU) complex, which mainly consists of four MCU subunits and essential MCU regulators (EMREs) [2, 46]. The regulatory proteins that interact with MCU subunits include mitochondrial calcium uptake 1/2 (MICU1/2) and MCU dominant-negative β-subunit (MCUb) [2, 46]. As its core component, the four MCU subunits were mainly in charge of mitochondrial calcium uptake and were upregulated by I/R injury, resulting in [Ca^2+^]_m_ overload and mitochondrial dysfunction [49]. In contrast, the inhibition of MCU alleviated cardiomyocyte apoptosis and myocardial reperfusion injury [15, 49]. The regulation and contribution of MCU activity was also highlighted in endothelial cells, and has been proven to be involved in ischemic microvascular diseases and atherosclerotic vascular disease [1]. Previous work suggested that the inhibition of MCU in the endothelium restrained [Ca^2+^]_m_ uptake and thereby improved coronary microvascular function in the face of I/R injury [27]. However, the detailed mechanisms by which the MCU complex regulates mitochondrial function and microvascular protection have not been fully explored.

Histidine triad (HIT) nucleotide-binding protein 2 (HINT2) is located in mitochondria and belongs to the HINT branch of the HIT superfamily [5]. Genetic ablation of HINT2 in mice resulted in oxidative respiratory chain impairment and highly acetylated mitochondrial proteins, which resembled the pattern of mitochondrial injury after hypoxia [33]. Our previous work verified that HINT2 overexpression preserved mouse heart function after MI, and maintained mitochondrial membrane potential (MMP) and mitochondrial respiration in cardiomyocytes exposed to hypoxia [11]. In addition, there is evidence that HINT2 can modulate intracellular calcium ([Ca^2+^]_i_) and [Ca^2+^]_m_ dynamics in hepatocytes and enhance apoptosis in pancreatic cancer cells by regulating the MCU complex [6, 35]. However, the exact role of HINT2 in I/R-induced cardiac microvascular injury has not been reported, and whether HINT2 can govern [Ca^2+^]_m_ fluctuation via the MCU complex and thereby improve mitochondrial dynamics and function in endothelial cells remains unknown. Therefore, the present study was designed to evaluate the effect of HINT2 overexpression on coronary microvascular protection in the setting of cardiac I/R injury and to explore the possible mechanisms with a focus on mitochondrial dynamics and MCU complex-related [Ca^2+^]_m_ homeostasis.

## Methods

### Endothelial HINT2 overexpression strategies

Mouse HINT2-overexpressing adeno-associated virus-9 (AAV9-HINT2) genome particles containing the TIE promoter and Flag were obtained from Genomeditech (China, Shanghai). To establish an endothelial cell (EC)-specific HINT2-overexpressing mouse model, 100 µl AAV9-HINT2 particles or negative control (AAV9-NC) at a density of 5 × 10^12 v.g./ml was injected into 6-week-old C57BL/6J mice via the caudal vein. Four weeks after transfection, cardiac tissues were sectioned to measure transfection efficiency of AAV9 in the cardiac microcirculation via co-immunofluorescence staining of Flag, cTnT, and CD31. Cardiac microvascular endothelial cells (CMECs) were isolated to measure HINT2 overexpression efficiency by Western blot.

Adenoviruses (ADVs) containing the HINT2 gene (ADV-HINT2) and a negative control (ADV-NC) were obtained from Hanbio (China, Shanghai). To establish HINT2-overexpressing CMECs in vitro, CMECs were isolated from 6- to 8-week-old C57BL/6J mice and transfected with ADV-HINT2 or ADV-NC at different multiplicities of infection (MOIs). HINT2 overexpression efficiency was determined using Western blot. Plasmids containing the full-length HINT2, the HINT2 mutant and the negative control (NC) were obtained from Genomeditech (China, Shanghai) and transfected into CMECs with Lipofectamine 3000 (Thermo Fisher, USA).

### Cardiac I/R injury model

Four weeks after AAV9-HINT2 transfection, male C57BL/6J mice (10 weeks of age) underwent surgical cardiac I/R injury as described previously [12]. The left anterior descending coronary artery (LAD) was ligated via a slipknot using 6–0 silk for 45 min, and then, the slipknot was gently loosened to induce reperfusion injury. Mice in the sham group underwent the same procedures without tying the slipknot. To inhibit the MCU complex, 50 nmol/kg Ru360 (MCE, USA) was injected intraperitoneally into AAV9-NC mice 1 h before I/R injury [15]. Spermine (5 mg/kg, MCE, USA) was injected intraperitoneally into AAV9-HINT2 mice 1 h before I/R injury to activate the MCU complex [27].

### CMECs isolation, oxygen–glucose deprivation/reoxygenation (OGD/R) injury, and cell viability assays

CMECs were isolated from the left ventricle according to a previous method [24]. Briefly, after the left ventricle were sufficiently digested into single cell suspension with liberase (Roche, Switzerland), CMECs were gathered using CD31-coupled microbeads (Thermo Fisher, USA) and further cultured in endothelial culture medium (ECM, ScienCell, USA) containing endothelial cell growth supplements (ECGS), 5% fetal bovine serum (FBS), 100 U/ml penicillin, and 100 μg/ml streptomycin. The purity of isolated CMECs was identified by immunofluorescence staining of CD31.

To induce OGD/R injury, CMECs were cultured in Eagle’s solution (Genom, China) and maintained in a hypoxia chamber (MGC, Japan) with a gas mixture of 5% CO_2_ and 95% N_2_ at 37 °C for 6 h to induce OGD injury. Reoxygenation injury was triggered by restoring the culture medium and atmosphere (5% CO_2_ and 95% air). Cells transfected with ADV-NC were pretreated with 10 μM Ru360 1 h before OGD/R injury to inhibit the MCU complex. Mdivi-1 (5 μM, MCE, USA) was added to the ADV-NC group during OGD/R injury to inhibit mitochondrial fission. In contrast, 1 μM FCCP (MCE, USA) or 10 μM spermine was applied to ADV-HINT2 cells for 2 h to reintroduce mitochondrial fission or reactivate the MCU complex, respectively. Cell viability was detected by a Cell Counting Kit-8 assay kit (EpiZymi, China).

### Thioflavin S staining assays

Thioflavin S staining assay was used to measure the myocardial no-reflow phenomenon [30, 44]. Briefly, 6% thioflavin S (1 ml/kg, MCE, USA) was injected into mice via the tail vein and allowed to circulate for 1–2 min. The hearts were quickly harvested, fixed in 4% paraformaldehyde (PFA) overnight, cut into 1 mm sections, and then observed under a stereomicroscope (Leica, Germany). 359 nm excitation light was used for imaging. The reflow area showed bright blue fluorescence, whereas the no-reflow area was dark. The no-reflow degree was indicated as the percentage of dark area to the left ventricle.

### Triphenyltetrazolium chloride (TTC) staining

TTC staining was conducted to measure cardiac infarction size [4]. After the mice were anesthetized with 2% isoflurane, the slipknot was tied again, and 1% Evans blue (w/v, Sigma, USA) was injected into the aortic root to perfuse the left ventricle. Then, the heart was rapidly excised, cut into 1 mm slices, and incubated with 1% TTC solution (Sigma, USA) at 37 °C for 10 min. Consecutive slices in each sample were scanned by a white light scanner (Canon, Japan). Area at risk (AAR) was defined as tissues not perfused by Evans blue. Infarcted myocardium was white, whereas viable myocardium was red. The infarction degree was calculated as the ratio of the infarction area to the AAR.

### Modified Miles assays

Evans blue (1%, w/v, 100 μl) was injected into mice via the tail vein. The mice were sacrificed 30 min after injection, and the hearts were perfused with citrate buffer (pH 4.5) through the aorta to evacuate blood. Left ventricle samples were immersed in 1 ml formamide, homogenized, and incubated at 60 °C overnight to elute Evans blue. The samples were centrifuged at 10,000×*g* for 40 min to extract the supernatant containing Evans blue. Absorbance at 620 nm was read by a FlexStation 3 instrument (Molecular Devices, USA). The concentration of Evans blue was calculated from a standard curve and corrected by left ventricle weight.

### Immunofluorescence staining

Frozen sections (6 μm) were fixed with cold acetone for 10 min at – 20 °C, air dried for 15 min, and blocked with 5% bovine serum albumin (BSA) for 1 h at room temperature (RT). The sections were then incubated with primary antibodies. After overnight incubation at 4 °C, the samples were washed with PBS and incubated with fluorescence-labeled secondary antibodies for 1 h at RT. To measure microvascular perfusion, 100 μl FITC-combined tomato lectin (1 mg/ml, Sigma, USA) was injected into mice via the tail vein and allowed to circulate for 10 min [31, 42]. Then, heart samples were harvested, sectioned, and stained for CD31 to label vessels. The microvascular perfusion ratio was indicated as the percentage of lectin-positive microvessels to CD31-positive microvessels. Immunofluorescence staining of cell samples was performed with the same procedures, except the samples were fixed with 4% PFA for 10 min and permeabilized with 0.5% Triton X-100 for 10 min. Detailed information of the primary and secondary antibodies used in immunofluorescence staining, including source, catalog number, and dilution, is listed in Table S1. All tissue sections and confocal dishes were observed under a confocal microscope (Olympus FV3000, Japan). At least five random fields were captured and quantitatively analyzed via ImageJ (version 1.53c, NIH, USA).

### Cell fluorescence staining

Mitochondria were stained with MitoTracker^®^ Deep Red FM (200 nM, Invitrogen™, USA) for 30 min in the dark. Cellular ROS and mitochondrial ROS (mtROS) were measured by CellROX^®^ Deep Red Reagent (5 μM, Invitrogen™, USA) and MitoSOX™ Red Mitochondrial Superoxide Indicator (5 μM, Invitrogen™, USA) for 30 min, respectively. To determine mitochondrial membrane potential (MMP), the cells were incubated with 2 µM JC-1 probe (Beyotime, China) for 30 min. MMP was calculated as the ratio of red-to-green fluorescence intensity according to the instructions. Cells were fixed, permeabilized, and then incubated with TUNEL (Sigma-Aldrich, USA) reaction mixture buffer for 60 min to detect apoptosis. [Ca^2+^]_i_ and [Ca^2+^]_m_ levels were measured with 5 μM Fluo-4 and 1 μM Rhod-2 (Thermo Fisher, USA), respectively, for 30 min at 37 °C. Nuclei were labeled by DAPI (Beyotime, China). All the above fluorescence images were captured with a laser confocal microscope (Olympus FV3000, Japan). Fluorescence intensity was analyzed with ImageJ (Version 1.53c, NIH, USA). Mitochondrial length was measured by MiNA (https://github.com/ScienceToolkit/MiNA), a plug-in in ImageJ.

### Nitrite detection

The nitrite content in cardiac tissue and cell culture medium was measured by a Micro NO Content assay kit (Solarbio, China) according to the principle of detection. Cardiac nitrite content was standardized to the protein concentration of smashed cardiac tissue.

### Migration, wound healing, and capillary tube formation assays

Migration assays were performed using Corning Transwell chambers (8 µm pores, 6.5 mm diameter, USA) that were precoated with 10 µg/ml fibronectin (ScienCell, USA). After OGD/R injury, cells were digested and seeded in the upper chambers containing FBS-free ECM. ECM containing 10% FBS was then added to the lower chambers to stimulate migration. After 24 h of culture, cells that traversed through the membrane were fixed with 4% PFA for 30 min and stained with 0.05% crystal violet solution (Solarbio, China) for 20 min. A wound-healing assay was performed by scratching the cell monolayer with a 200 µl pipette tip in 6-well plates. Cells were washed with PBS twice and cultured in ECM without FBS. Images were collected at the beginning and 48 h after scratching. The wound-healing speed was calculated as the ratio of the healed areas at 48 h to the original injured areas. A capillary tube formation assay was performed using the following procedures. Matrigel (50 µl, BD Bioscience) was added to each well of a 96-well plate and solidified at 37 °C for 30 min. CMECs were digested and resuspended in ECM. A total of 5 × 10^4^ cells were seeded in each well and cultured at 37 °C for 3 h. Capillary-like tubes were imaged by an optical microscope (Leica DM3000, Germany), and the numbers of branch points were counted manually.

### Endothelial monolayer permeability assays

CMECs (1 × 10^5^) were seeded in the upper chamber of a Corning Transwell (0.4 μm pore size, 6.5 mm diameter, USA) that was precoated with fibronectin. After OGD/R injury, 100 μl FITC-BSA (68 kDa, Sigma, USA) was added to the upper chamber at a concentration of 1 mg/ml. After 1 h of free permeation, the fluorescence intensity of FITC-BSA in the upper and lower chambers was quantified by a FlexStation 3 (Molecular Devices, USA).

### Protein sample preparation and Western blotting assay

Total proteins were extracted in RIPA lysis buffer (Beyotime, China) on ice, followed by centrifugation at 12,000 rpm for 30 min to remove cell fragments. Mitochondria were extracted by lysing cells with Mitochondrial Separation Reagent (Beyotime, China) on ice for 15 min. After centrifugation, mitochondria were separated from cytoplasmic fragments and further lysed by Mitochondrial Lysis Buffer to extract mitochondrial protein. For immunoprecipitation (IP), cells were lysed with Cell Lysis Buffer for IP (Weiao Biological Company, China) on ice for 15 min and incubated with protein A/G-magnetic beads conjugated with antibodies against VE-Cadherin (Abcam, ab205336) and Flag (Abcam, ab205606) for 30 min at RT under constant rotation. Rabbit IgG (Abcam, ab172730) was used as a technical negative control to assess nonspecific binding. The immunocomplexes were mixed with loading buffer and boiled for 10 min to elute the target protein. Nondenatured protein samples were obtained by lysing cells with Native Lysis Buffer and mixing with Native-PAGE loading buffer (Solarbio, China) without heating. Carbonylated mitochondrial proteins were detected by a protein oxidation detection kit (Sigma, USA). After extraction, mitochondrial proteins were incubated with 2.4-dinitrophenylhydrazine (DNPH) to conjugate carbonyl groups.

After quantified, protein samples (10–20 μg) were loaded on SDS-polyacrylamide gels for electrophoresis and then transferred to PVDF membranes. After blocking with 5% BSA for 1 h, the membranes were incubated with primary antibodies overnight at 4 °C. Later, after rinsing with TBST for three times, the membranes were incubated with horseradish peroxidase (HRP)-conjugated secondary antibodies (Weiao Biological Company, China) at RT for 1 h. The blots were shown by Pierce™ enhanced chemiluminescence (ECL) Western blotting Substrate (Thermo Fisher, USA). The band intensities were analyzed by ImageJ (Version 1.53c, NIH, USA). Native-PAGE was performed for the detection of DRP1 oligomers according to the same procedure except nondenatured protein samples (10 μg) were loaded on polyacrylamide gels without denaturants (EpiZyme, China). Detailed information of the primary antibodies used in immunoblotting, including source, catalog number, and dilution, is listed in Table S2.

### Structure-based protein interaction interface analysis

The protein structures were predicted by Modeller (salilab.org/modeller) using Protein Data Bank (PDB) structure 6YI0 as template for HINT2 and 6o58 as template for MCU. Structures of proteins were submitted to the zdock tool (http://zdock.umassmed.edu/) to predict potential amino acid (AA) binding sites. Binding free energy (BFE) between proteins was calculated by MMGBSA analysis.

### Statistical analysis

The data are expressed as the means ± SEMs. The Shapiro–Wilk test and Kolmogorov–Smirnov test were performed before parametric tests, and the results showed that all obtained data were normally distributed. Statistical analyses were performed with Student’s *t* test or one-way analysis of variance (ANOVA) followed by post hoc tests. *P* < 0.05 was considered statistically significant.

## Results

### Endothelial-specific overexpression of HINT2 alleviated cardiac microvascular dysfunction after I/R injury

**Fig. 1:**
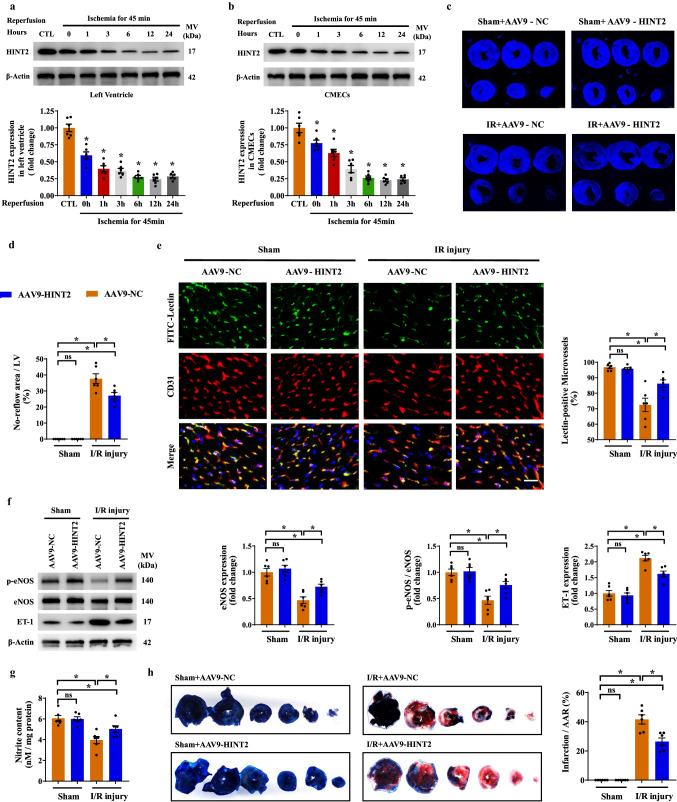
Endothelial-specific overexpression of HINT2 alleviated cardiac microvascular injury and myocardial infarction after I/R injury. After 45 min of ischemia and 0–24 h of reperfusion, the expression of HINT2 in left ventricle and isolated CMECs was measured by Western blot analysis (**a**, **b**). **c**–**h** Mice were transfected with AAV9-NC or AAV9-HINT2 and suffered from 45 min ischemia injury and 6 h reperfusion injury. No reflow area was detected by thioflavin S staining and quantified (**c**, **d**). Cardiac microvascular perfusion was indicated by the ratio of lectin-positive microvessels (green) to CD31-positive microvessels (red). Scale bars: 15 μm (**e**). eNOS expression, eNOS phosphorylation at Ser^1177^, and ET-1 expression in left ventricle were detected by Western blot analysis (**f**). Nitrite content in left ventricle (**g**). Infarcted area was detected by TTC staining and quantified (**h**). *P < 0.05, significantly different as indicated. *ns* Not significant. Biological replicates were performed for six times in the above studies

In our mouse model of cardiac I/R injury, HINT2 was significantly reduced in both cardiac tissues and primary CMECs in ischemic region after 45 min of ischemia and reached the lowest point after 6 h of reperfusion (Fig. 1a, b). Therefore, mice suffering 6 h of reperfusion injury were selected as our following study subjects. To gain more insight into the role of HINT2 in microvascular protection, an AAV9-mediated endothelial-specific HINT2 overexpression mouse model was established. Fluorescence staining of Flag indicated the transfection efficiency of AAV9 was > 95% in the microcirculation (Fig. S1a), and HINT2 signal was higher in the Flag-positive area in AAV9-HINT2-infected hearts (Fig. S1b). Meanwhile, HINT2 expression in isolated CMECs was ~ 2.82-fold higher than that in mice transfected with AAV9-NC (Fig. S1c). Moreover, HINT2 was not transfected into cardiomyocytes, as no obvious colocalization between Flag and cTnT was observed (Fig. S1a), and the expression of HINT2 was equal in cardiomyocytes isolated from the AAV9-NC and AAV9-HINT2 groups (Fig. S1d).

To examine the impact of HINT2 on microvascular damage, the no-reflow phenomenon was measured. An obvious perfusion defect of thioflavin S was observed in the left ventricle after reperfusion, and HINT2 overexpression significantly reduced the area of no-reflow (Fig. 1c, d). The fundamental pathology of the no-reflow phenomenon was highly related to compromised microvascular perfusion. In our mouse model, the microvasculature became sparse after reperfusion and was accompanied by a lower ratio of lectin-positive microvessels, suggesting decreased microvascular perfusion (Fig. 1e). In contrast, HINT2 overexpression obviously relieved perfusion defects (Fig. 1e). Microvascular patency is highly dependent on endothelium-controlled vasodilatation. Nitrite content, eNOS expression, and eNOS phosphorylation at Ser^1177^, a biologically active site, were significantly downregulated in ischemic ventricles after I/R injury, and the vascular constriction factor ET-1 was markedly increased (Fig. 1f, g). The above effects were obviously alleviated by HINT2 transfection (Fig. 1f, g). Further works revealed that I/R injury led to microvascular barrier collapse, as evidenced by higher Evans blue leakage in ischemic tissues (Fig. S1e). However, HINT2 overexpression significantly inhibited Evans blue permeation (Fig. S1e). Besides, VE-Cadherin were reduced in I/R injury, while a possible improvement of VE-Cadherin with HINT2 overexpression was observed, although the presented quantification still suggested a significant reduction compared to sham animals (Fig. S1f). Moreover, HINT2 overexpression reduced VE-Cadherin phosphorylation at Tyr^731^, a binding site for β-Catenin, and, correspondingly, enhanced the interaction between VE-Cadherin and β-Catenin during I/R injury (Fig. S1f, g). Ultimately, endothelial-specific overexpression of HINT2 reduced the infarction size (Fig. 1h), indicating that HINT2-mediated microvascular improvement inhibited myocardial necrosis in the acute reperfusion phase.

### HINT2 overexpression balanced mitochondrial dynamics in CMECs both in vivo and in vitro

**Fig. 2:**
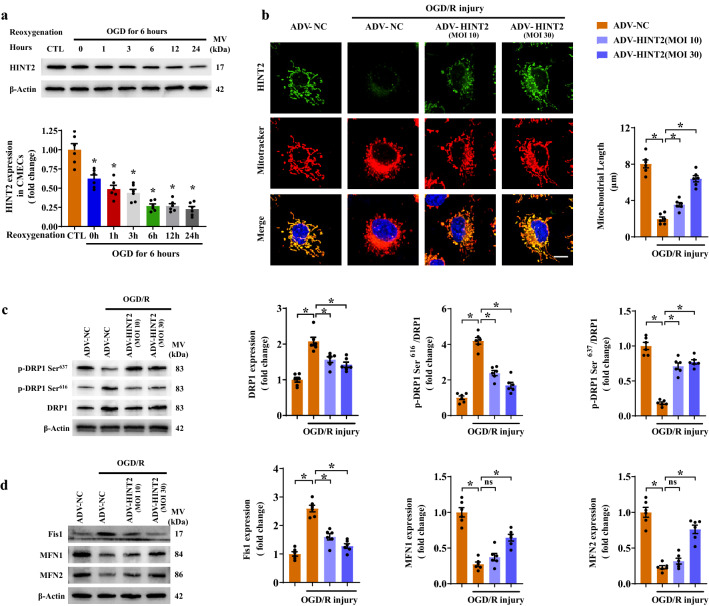
HINT2 overexpression balanced mitochondrial dynamics in CMECs suffering from OGD/R injury. CMECs were isolated from 6- to 8-week-old WT mice and subjected to 6 h of OGD injury and 0–24 h of reoxygenation injury; the expression of HINT2 was measured by Western blot analysis (**a**). **b**–**d** Isolated CMECs were transfected with ADV-NC or ADV-HINT2, and then subjected to 6 h of OGD injury and 6 h of reoxygenation injury. HINT2 expression and mitochondrial morphology were evaluated by immunofluorescence staining, and mitochondrial length was analyzed. Scale bars: 10 μm (**b**). DRP1 expression, DRP1 phosphorylation at Ser^616^, and DRP1 phosphorylation at Ser^637^ were detected by Western blot analysis (**c**). Western blot analysis of the protein expression of Fis1, MFN1 and MFN2 (**d**). *P < 0.05, significantly different as indicated. *ns* Not significant. Biological replicates were performed for six times in the above studies

To elucidate the potential mechanism by which HINT2 protected the cardiac microvasculature against reperfusion injury, the present work focused on mitochondrial dynamics, an important perspective in cardiac microvascular diseases [7, 9]. Mitochondrial dynamics-related molecules were monitored in CMECs isolated from I/R models. After reperfusion injury, DRP1 and Fis1 were obviously upregulated in isolated CMECs, whereas MFN1 and MFN2 was notably reduced, indicating that mitochondrial fission may be enhanced (Fig. S2a, b). In comparison, the above molecular changes were reversed by HINT2 overexpression (Fig. S2a, b). To further clarify the mechanisms underlying HINT2-mediated mitochondrial dynamics, in vitro studies were performed by challenging CMECs with OGD/R injury. HINT2 expression gradually decreased during the reoxygenation stage and reached its lowest point within 6 h (Fig. 2a). Therefore, 6 h of reoxygenation injury following OGD was selected to simulate endothelial I/R injury in vitro. Fluorescence staining showed the mitochondrial net became short and fragmented after OGD/R injury, whereas ADV-HINT2 transfection at 10 and 30 MOI maintained mitochondrial length and morphology (Fig. 2b). Further works confirmed that HINT2 transfection at 30 MOI modified dynamics-related molecules after OGD/R injury, as evidenced by reduced DRP1 and Fis1 expression, inhibited DRP1 oligomerization and DRP1 phosphorylation at Ser^616^, increased MFN1 and MFN2 expression, and enhanced DRP1 phosphorylation at Ser^637^ (Fig. 2c, d and Fig. S2c). Similar results were observed in the 10 MOI-transfected cells, except the reduction in MFN1 and MFN2 was not rescued (Fig. 2c, d and Fig. S2c), indicating that HINT2 overexpression was more prone to alleviate DRP1/Fis1-regulated mitochondrial fission. Then, gain- and loss-of-function assays of mitochondrial fission were conducted. Mdivi-1, a DRP1-specific inhibitor, increased mitochondrial length and cell viability, whereas FCCP, a fission enhancer, abolished the beneficial actions of HINT2 in sustaining the mitochondrial network and cell survival (Fig. S2d, e). Altogether, the above data demonstrated that HINT2 is necessary for balancing mitochondrial dynamics in CMECs suffering reperfusion/reoxygenation injury.

### HINT2-mediated inhibition of mitochondrial fission protected CMECs against reoxygenation-induced mitochondrial dysfunction

**Fig. 3:**
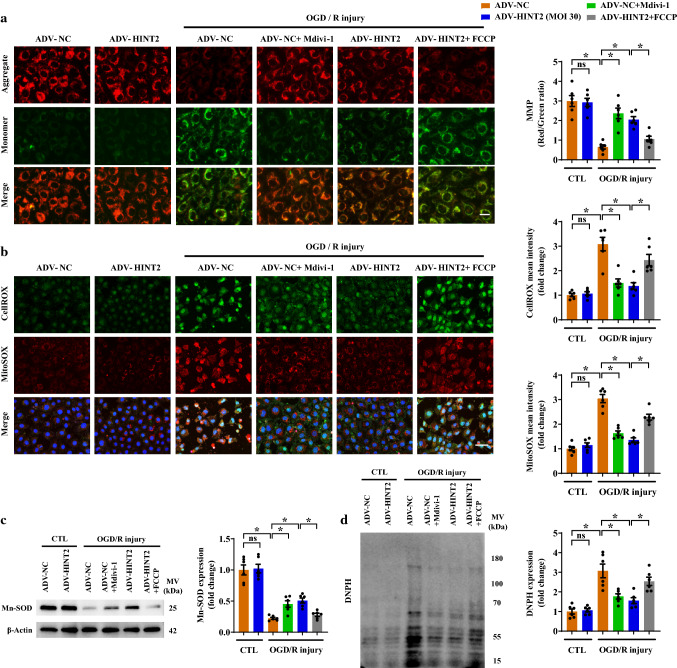
HINT2 overexpression alleviated OGD/R injury-induced mitochondrial dysfunction by restraining mitochondrial fragmentation. CMECs were isolated from 6- to 8-week-old WT mice, transfected with ADV-NC or ADV-HINT2, and subjected to OGD/R injury. Mdivi-1 (5 μM) was added to the ADV-NC group during OGD/R injury to inhibit mitochondrial fission. FCCP (1 μM) was applied to ADV-HINT2 cells during OGD/R injury for 2 h to reintroduce mitochondrial fission. Mitochondrial membrane potential was measured using JC-1 fluorescence probe. Scale bars: 20 μm (**a**). Cellular ROS and mitochondrial ROS were stained with CellROX (green) and MitoSOX (red), respectively. Scale bars: 60 μm (**b**). Mn-SOD expression was detected by Western blot analysis (**c**). Protein lysates were incubated with DNPH to label carbonyl groups, and protein carbonylation was detected by Western blot analysis using anti-DNP antibodies (**d**). *P < 0.05, significantly different as indicated. *ns* Not significant. Biological replicates were performed for six times in the above studies

Attempts were then made to identify whether the inhibitory effects of HINT2 on mitochondrial fission would protect mitochondrial function. Depolarized MMP is an early marker of mitochondrial dysfunction and apoptosis. MMP was significantly reduced after OGD/R injury, whereas HINT2 overexpression or Mdivi-1 treatment alleviated this reduction (Fig. 3a). However, regaining fission with FCCP antagonized the protective effects of HINT2 on MMP collapse (Fig. 3a). OGD/R injury resulted in a mitochondrial ROS (mtROS) burst and cellular ROS accumulation, whereas ADV-HINT2 transfection or Mdivi-1 treatment suppressed the mtROS burst and cellular ROS accumulation (Fig. 3b). Mn-SOD is one of the most forceful ROS scavengers in mitochondria. After OGD/R injury, the expression of Mn-SOD was significantly reduced, and this effect was alleviated by HINT2 overexpression or Mdivi-1 treatment (Fig. 3c). In addition, HINT2 overexpression or Mdivi-1 treatment suppressed the carbonyl modification of mitochondrial proteins caused by ROS toxicity (Fig. 3d). It can be predicted that regaining fission abrogated the suppressive effects of HINT2 on ROS accumulation, Mn-SOD down-regulation, and mitochondrial protein carbonylation (Fig. 3b–d). Taken together, these data confirmed that the enhanced mitochondrial function induced by HINT2 was highly associated with its role in inhibiting mitochondrial fission.

### Overexpression of HINT2 suppressed mitochondrion-dependent apoptosis after OGD/R injury

**Fig. 4:**
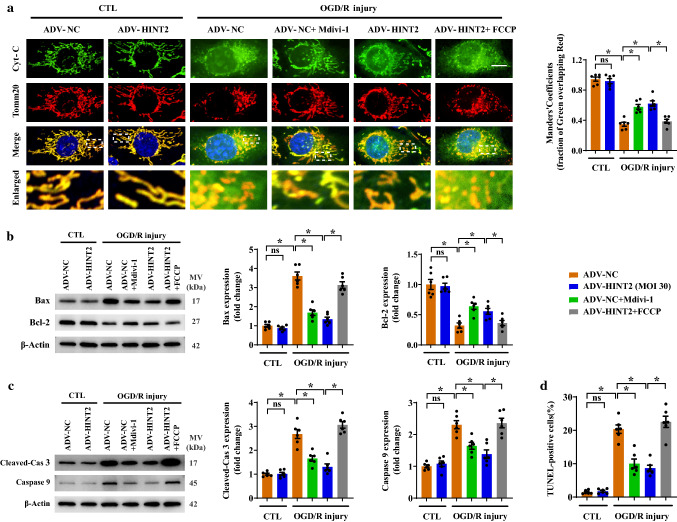
HINT2 overexpression inhibited the mitochondrion-dependent apoptosis signaling pathway activated by OGD/R injury. CMECs were isolated from 6- to 8-week-old WT mice, transfected with ADV-NC or ADV-HINT2, and subjected to OGD/R injury. Mdivi-1 (5 μM) was added to the ADV-NC group during OGD/R injury to inhibit mitochondrial fission. FCCP (1 μM) was applied to ADV-HINT2 cells during OGD/R injury for 2 h to reintroduce mitochondrial fission. The analysis of colocalization between mitochondria and cytochrome C was performed by immunostaining of cytochrome C (green) and Tomm20 (red). Cyt-C: cytochrome C. Scale bars: 10 μm (**a)**. Western blot analysis of the protein expression of Bax and Bcl-2 (**b**). Western blot analysis of the protein expression of cleaved-Caspase 3 and Caspase 9. Cleaved-Cas 3: cleaved-Caspase 3 (**c**). Cell apoptosis was detected by TUNEL staining and quantified (**d**). *P < 0.05, significantly different as indicated. *ns* Not significant. Biological replicates were performed for six times in the above studies

Enhanced mitochondrial fission resulted in cytochrome C release, which further activated the mitochondrion-dependent apoptosis pathway. After OGD/R injury, cytochrome C was released from mitochondria to the cytoplasm in CMECs, as evidenced by the decreased colocalization of mitochondria and cytochrome C (Fig. 4a). Inhibition of fission with Mdivi-1 or HINT2 overexpression restricted cytochrome C release (Fig. 4a). Following cytochrome C release, the expression of Bax was upregulated and Bcl-2 was downregulated (Fig. 4b), which was accompanied by enhanced expression cleaved-caspase 3 and caspase 9 (Fig. 4c), indicating that mitochondrion-dependent apoptosis was completely activated. In contrast, Mdivi-1 treatment or HINT2 overexpression suppressed the activation of the mitochondrion-dependent apoptosis pathway (Fig. 4b, c). Moreover, TUNEL-positive CMECs were reduced by Mdivi-1 treatment or HINT2 overexpression, indicating that OGD/R injury-induced apoptosis was suppressed (Fig. 4d and Fig. S2f). However, enhancing mitochondrial fission once more by FCCP abolished all the antiapoptotic effects of HINT2 elaborated above (Fig. 4a–d and Fig. S2f).

### HINT2 inhibited mitochondrial calcium inflow by directly binding MCU in CMECs under OGD/R injury

**Fig. 5:**
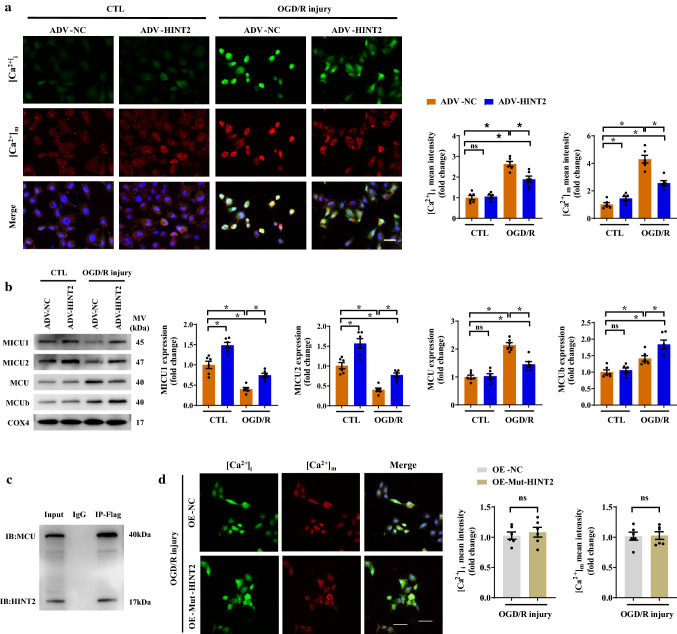
HINT2 maintained mitochondrial calcium homeostasis by directly binding the MCU complex. CMECs were isolated from 6- to 8-week-old WT mice, transfected with ADV-NC or ADV-HINT2, and subjected to OGD/R injury. [Ca^2+^]_i_ and [Ca^2+^]_m_ were stained with Fluo-4 AM (green) and Rhod-2 (red), respectively. Scale bars: 40 μm (**a**). Western blot analysis of the protein expression of MICU1, MICU2, MCU, and MCUb (**b**). Co-IP analysis of MCU and Flag-tagged HINT2 in CMECs (**c**). CMECs were transfected with plasmids containing HINT2 mutant or the negative control, and subjected to OGD/R injury. [Ca^2+^]_i_ and [Ca^2+^]_m_ were stained with Fluo-4 AM (green) and Rhod-2 (red), respectively. Mut-HINT2: HINT2 mutant. NC: negative control. Scale bars: 40 μm (**d**). *P < 0.05, significantly different as indicated. *ns* Not significant. Biological replicates were performed for 3–6 times in the above studies

Calcium overload is involved in I/R injury-induced mitochondrial fission/apoptosis, and HINT2 has been suggested to modulate [Ca^2+^]_m_ inflow. Therefore, we hypothesized that the suppressive effect of HINT2 on mitochondrial fission/apoptosis in endothelial cells was associated with [Ca^2+^]_m_ overload. First, our data indicated that lowering [Ca^2+^]_m_ exerted inhibitory effects on mitochondrial fission. Restricting [Ca^2+^]_m_ influx by Ru360, a specific MCU inhibitor, inhibited mitochondrial fission under OGD/R injury by suppressing DRP1 phosphorylation at Ser^616^ and DRP1 oligomerization (Fig. S3a–c). Then, further work demonstrated HINT2 overexpression significantly downregulated [Ca^2+^]_i_ and [Ca^2+^]_m_ levels under reoxygenation conditions (Fig. 5a). [Ca^2+^]_m_ homeostasis is largely dependent on the MCU complex. MCU was increased after reoxygenation injury but was reduced by HINT2 overexpression (Fig. 5b). In contrast, MICU1 and MICU2 were decreased after reoxygenation and maintained by HINT2 overexpression (Fig. 5b). Moreover, MCUb was significantly increased after reoxygenation, and HINT2 further enhanced MCUb expression (Fig. 5b). Co-IP assay showed that HINT2 could directly bind to MCU (Fig. 5c), and 4 AAs in HINT2 were identified as potential binding sites by structure-based protein interaction interface analysis (Fig. S3d). All AAs are within the domain of HINT2 but out of transit peptides that targeting mitochondria. Leu76, Phe78, and Asp80 are adjacent, while Trp160 is far from the other three AAs in spatial position. Based on the above results, Leu76, Phe78, and Asp80 were mutated into Ala in HINT2 mutant (Fig. S3e). MMGBSA analysis showed that HINT2 mutant had higher BFE with MCU than wild type (Fig. S3f). Therefore, a plasmid containing HINT2 mutant was constructed and transfected into CMECs. Although HINT2 mutant still colocalized with MCU in mitochondria (Fig. S3g), the direct interaction between HINT2 and MCU was interrupted, as shown by the Co-IP assay (Fig. S3h), suggesting that the binding site in HINT2 was located within Leu76, Phe78, and Asp80. Additionally, overexpression of HINT2 mutant failed to inhibit calcium overload and mitochondrial fragmentation in CMECs under OGD/R injury (Fig. 5d and Fig. S3i). Taken together, these data established that HINT2 stabilizes [Ca2^+^]_m_ homeostasis by directly binding MCU and modulating the MCU complex, thereby inhibiting excessive mitochondrial fission in CMECs suffering OGD/R injury.

### Overexpressing HINT2-alleviated CMEC functions after OGD/R injury by mitigating mitochondrial calcium overload

**Fig. 6:**
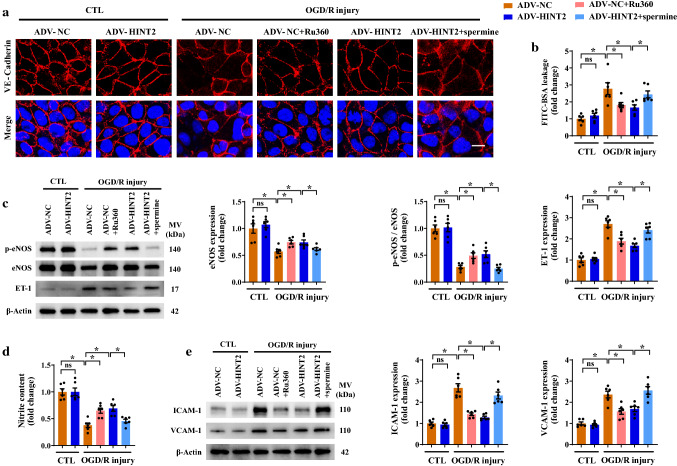
Enhancing mitochondrial calcium influx negated the protective effects of HINT2 on CMECs in vitro. CMECs were isolated from 6- to 8-week-old WT mice, transfected with ADV-NC or ADV-HINT2, and subjected to OGD/R injury. Cells transfected with ADV-NC were pretreated with Ru360 (10 μM) 1 h before OGD/R injury to inhibit the MCU complex. Spermine (10 μM) was added to ADV-HINT2-infected cells during OGD/R injury for 2 h to reactivate the MCU complex. Endothelial junction was shown by immunofluorescence staining of VE-Cadherin (red). Scale bars: 20 μm (**a**). Endothelial monolayer permeability was evaluated by FITC-BSA leakage assay (**b**). eNOS expression, eNOS phosphorylation at Ser^1177^, and ET-1 expression were detected by Western blot analysis (**c**). Nitrite content in culture medium (**d**). Western blot analysis of the protein expression of ICAM-1 and VCAM-1 (**e**). *P < 0.05, significantly different as indicated. *ns* Not significant. Biological replicates were performed for six times in the above studies

Attempts were then made to determine whether HINT2 improves CMEC functions via MCU and [Ca^2+^]_m_ inhibition. After OGD/R injury, CMEC migration and tube formation ability were obviously impaired, the effects of which were largely rectified by HINT2 overexpression or Ru360 (Fig. S4a–c). Endothelial migration and angiogenesis are mediated mainly by the VEGF pathway. Ru360 treatment or HINT2 overexpression enhanced VEGFR2 expression and further upregulated VEGF signaling in OGD/R-injured CMECs (Fig. S4d). However, these contributions of HINT2 to facilitate cell migration, angiogenesis, and VEGF pathway activation were largely abolished after enhancing [Ca^2+^]_m_ entry by spermine, an MCU activator (Fig. S4a–d). In addition, Ru360 treatment or HINT2 overexpression restrained the leakage of FITC-BSA from the impaired endothelial monolayer after OGD/R injury by enhancing VE-Cadherin junctions and VE-Cadherin/β-Catenin interaction (Fig. 6a, b and Fig. S4e). However, these beneficial effects of HINT2 on maintaining endothelial barrier function were completely negated after the reintroduction of excessive [Ca^2+^]_m_ (Fig. 6a, b and Fig. S4e). After OGD/R injury, eNOS expression, eNOS phosphorylation and nitrite content were decreased, indicating the potential for less NO to be released from CMECs (Fig. 6c, d). Ru360 treatment or HINT2 overexpression significantly increased nitrite content in culture medium, along with increased eNOS expression and eNOS phosphorylation (Fig. 6c, d). However, the beneficial effects of HINT2 on nitrite levels, eNOS expression, and eNOS phosphorylation were completely suppressed after spermine treatment (Fig. 6c, d). Moreover, spermine offset the inhibitory effects of HINT2 on ET-1 upregulation (Fig. 6c). Hypoxia plays a permissive role in motivating inflammatory response. Ru360 treatment or HINT2 overexpression decreased ICAM-1 and VCAM-1 expression in OGD/R-injured cells (Fig. 6e). It could be expected that the above anti-inflammatory actions of HINT2 were completely abolished after regaining [Ca^2+^]_m_ overload (Fig. 6e). In summary, the above results confirmed that HINT2 preserved angiogenic potential, strengthened endothelial barrier function, stimulated eNOS activation, and inhibited inflammatory response in CMECs exposed to reperfusion injury in vitro, at least in part due to its role in maintaining calcium homeostasis.

### Reactivation of the MCU complex abolished the protective effects of HINT2 overexpression on cardiac microvascular functions in I/R mice

**Fig. 7:**
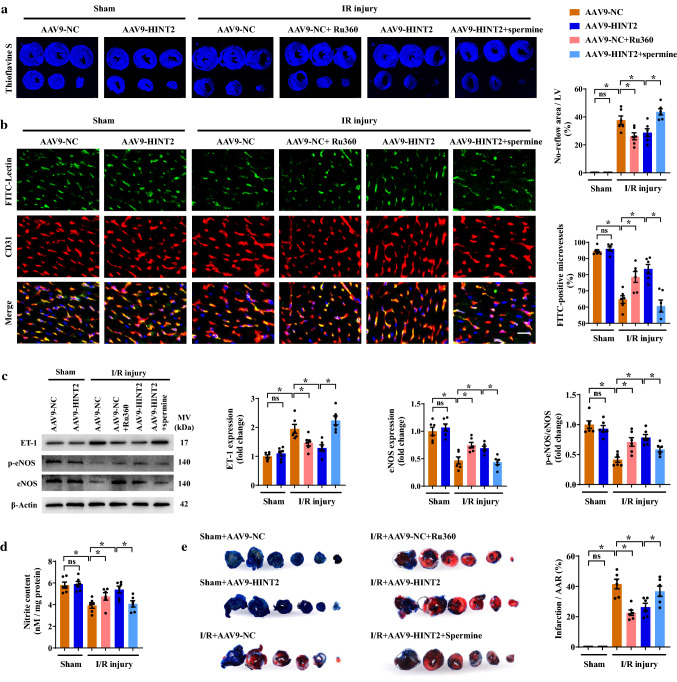
Endothelial-specific overexpression of HINT2 protected the cardiac microcirculation against I/R injury by modulating the MCU complex. Mice were transfected with AAV9-NC or AAV9-HINT2 and suffered from 45 min ischemia and 6 h reperfusion injury. Ru360 (50 nmol/kg) was injected intraperitoneally into AAV9-NC mice 1 h before I/R injury to inhibit the MCU complex. Spermine (5 mg/kg) was injected intraperitoneally into AAV9-HINT2 mice 1 h before I/R injury to activate the MCU complex. No reflow area after reperfusion was detected by thioflavin S staining and quantified (**a**). Cardiac microvascular perfusion was indicated by the ratio of lectin-positive microvessels (green) to CD31-positive microvessels (red). Scale bars: 20 μm (**b**). ET-1 expression, eNOS expression, and eNOS phosphorylation at Ser^1177^ in left ventricle were detected by Western blot analysis (**c**). Nitrite content in left ventricle (**d**). Infarcted area was detected by TTC staining and quantified (**e**). *P < 0.05, significantly different as indicated. *ns* Not significant. Biological replicates were performed for six times in the above studies

To gain more convincing evidence that the MCU complex was a downstream target of HINT2 in cardiac microvascular protection, the effects of Ru360 and spermine were investigated in mice. Similar to HINT2 overexpression, Ru360 treatment alleviated the no-reflow phenomenon and maintained microvascular perfusion in ischemia hearts, as evidenced by increased thioflavin S perfusion, more FITC-perfused microvessels, enhanced eNOS phosphorylation, suppressed ET-1 expression, and higher nitrite content (Fig. 7a–d). However, in the presence of spermine treatment, HINT2 overexpression failed to alleviate the no-reflow phenomenon and enhance microvascular perfusion (Fig. 7a–d). As we have shown previously, HINT2 overexpression reduced VE-Cadherin phosphorylation and strengthened the VE-Cadherin/β-Catenin interaction, thereby inhibiting Evans blue leakage from cardiac microcirculation (Fig. S5a–c). However, these improvements were not observed in the AAV-HINT2 group pretreated with spermine (Fig. S65-c). Moreover, spermine-mediated MCU complex activation abrogated the suppressive effects of HINT2 on VCAM-1 and ICAM-1 expression (Fig. S5d). Importantly, blocking the MCU complex with Ru360 limited the myocardial infarction size, whereas activating the MCU complex nullified the protective effects of HINT2 on infarction expansion (Fig. 7e). In conclusion, the above in vivo data further explicitly showed that the protective effects of HINT2 relied on the inhibition of the MCU complex and [Ca^2+^]_m_ overload.

## Discussion

**Scheme 1:**
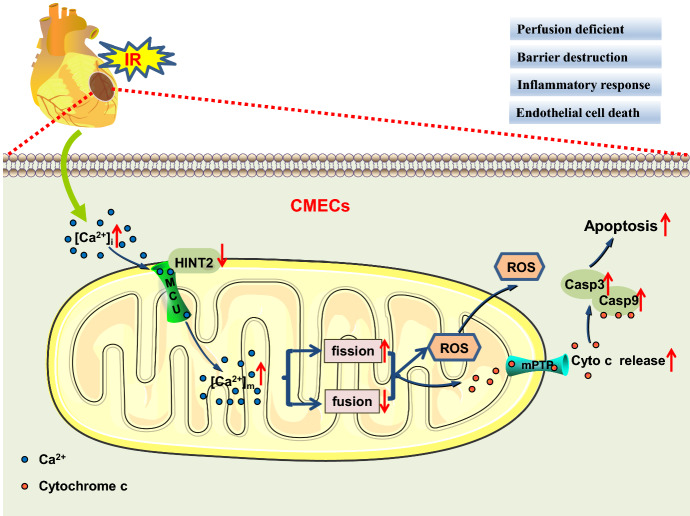
Diagram of potential mechanism for HINT2 in repairing I/R-induced cardiac microvascular injury

The cardiac microcirculation is particularly vulnerable to the detrimental effects of reperfusion and has been recognized as a therapeutic target for cardiac I/R injury. The present data demonstrated that HINT2 overexpression protected the cardiac microcirculation against I/R injury by enhancing blood perfusion, barrier function, and anti-inflammatory action. The protection from HINT2 was attributed to its role in regulating MCU complex function and maintaining [Ca^2+^]_m_ homeostasis in CMECs. HINT2 overexpression inhibited [Ca^2+^]_m_ overload-evoked mitochondrial fission and, thereby, maintained mitochondrial function, and inhibited the mitochondrion-dependent apoptotic pathway, ultimately improving endothelial survival and function in response to reoxygenation injury (Scheme 1).

It is widely acknowledged that a timely reperfusion remedy is necessary for rescuing ischemic myocardium [22]. In contrast, after reperfusion, superabundant reactive species and inflammatory cascades burst rapidly and assault the cardiac microcirculation [29]. The most immediate consequence is cardiac edema and, if severe, slow-reflow and no-reflow phenomena, which discounted the benefits of coronary reperfusion strategies in MI patients [17]. The present study identified that HINT2 overexpression enhanced CMEC migration and angiogenesis by mediating the VEGF signaling pathway, which sustained CMEC survival in the setting of reoxygenation. In addition, HINT2 overexpression increased nitrite content, eNOS expression, and phosphorylation in the myocardium and CMECs, along with decreased ET-1 expression, indicating a protective role for HINT2 in the cardiac vasodilator response. Through the above mechanisms, HINT2 maintains microvascular density and increases blood reperfusion in the ischemic region. During the reperfusion stage, increased microvascular permeability and inflammatory exudation are the major contributors to cardiac edema. HINT2 overexpression strengthened VE-Cadherin/β-Catenin interaction and suppressed VCAM-1 and ICAM-1 to enhance endothelial barrier function and prevent inflammatory response in both I/R-injured myocardium and OGD/R-injured CMECs. In combination with our previous study, these newly obtained data further support the beneficial role of HINT2 in cardioprotection, especially in microvascular structure and function during the reperfusion stage.

Long-term and substantially elevated [Ca^2+^]_m_ triggered MMP collapse, ROS overproduction, mPTP opening, and, most importantly, apoptosis in cardiomyocytes, which was identified as a core mechanism underlying cardiac I/R injury [40]. However, the pathological role of [Ca^2+^]_m_ in mitochondrial fission and endothelial functions in I/R injury was not explored in detail. The present study revealed that the restoration of [Ca^2+^]_m_ by Ru360 inhibited mitochondrial fission caused by OGD/R injury via inhibition of DRP1 phosphorylation and oligomers in CMECs. Moreover, fission inhibition favored the recovery of mitochondrial dysfunction and the suppression of the mitochondrial apoptosis pathway in OGD/R-injured CMECs. A few studies have been performed and proposed that the suppression of [Ca^2+^]_m_ overload maintains endothelial functions under I/R injury, hyperuricemia, and diabetes [20, 37, 45]. The present study demonstrated that the inhibition of [Ca^2+^]_m_ overload via HINT2 overexpression or Ru360 treatment protected CMECs from reoxygenation injury in terms of endothelial barrier collapse, eNOS reduction, inflammatory reaction, and angiogenic disorder. Additionally, our further attempts confirmed that the maintenance of [Ca^2+^]_m_ homeostasis relieved cardiac microvascular dysfunction induced by I/R injury, as evidenced by decreased no-reflow area, increased reperfusion, enhanced barrier function, and reduced expression of adhesion molecules, all of which indispensably contributed to limiting infarction expansion.

[Ca^2+^]_m_ influx is principally mediated by the MCU complex, which is critical for mPTP opening in response to increased [Ca^2+^]_I_ [2]. The identification of subunits in the MCU complex paves the way to determine the different roles of each subunit in cardiac I/R injury [24]. Four MCU subunits constitute the basic components of MCU complexes. Cardiomyocyte-specific deletion of MCU subunits or Ru360 treatment protected cardiomyocytes from [Ca^2+^]_m_ overload and mPTP opening and, therefore, decreased infarct size and preserved cardiac function in I/R hearts [15, 43]. Importantly, MCU inhibition in CMECs has been suggested to improve cardiac microcirculation after I/R injury [27]. MICU1 and MICU2 form heterodimers and act as MCU gatekeepers to keep the channel closed by sensing the [Ca^2+^]_i_ concentration [38]. Restraining [Ca^2+^]_m_ influx via the upregulation of MICU1 protected the heart from I/R injury [3, 50]. MCUb was not expressed at baseline, but was upregulated when sensing calcium elevation in the cytoplasm [21]. MCUb overexpression reduced mPTP opening and decreased infarct size following IR injury [21, 26]. Although the function of MCU in the cardiac microcirculation was investigated before, other subunits were ignored in this research field. HINT2 was introduced only to modulate the MCU complex in pancreatic cancer, sensitizing tumor cells to apoptosis by enhancing [Ca^2+^]_m_ uptake [6]. The current work showed that HINT2 overexpression inhibited the upregulation of MCU, reversed the reduction in MICU1 and MICU2, and further increased MCUb expression in CMECs under OGD/R injury. A protein–protein interaction study identified Leu76, Phe78, and Asp80 as sites for HINT2 to directly combine with MCU in CMECs. The above results indicated that HINT2 exerted protective benefits on endothelial cells by directly binding MCU and thereby regulating the MCU complex and [Ca^2+^]_m_ homeostasis in the setting of reoxygenation injury.

Several potential biases or imprecisions should be considered. Unspecific transfection of AAV9-HINT2 had only been excluded in cardiomyocytes but not in other cell types, such as fibroblasts and smooth muscle cells. Smooth muscle cells are major components of arterioles and are in charge of vasodilatation. Based on the presented data, a role of HINT2 in vascular physiology is sensible, but the contribution from other cell types cannot be excluded. The present study used the FITC-lectin perfusion method as a surrogate parameter to estimate microvascular blood flow [31, 42]. More direct methods to determine coronary flow and reactive hyperemia of the coronary arteries during reperfusion would provide more precise conclusions [16, 23]. The short half-life of NO makes it difficult to directly detect NO content in the myocardium [32]. The present study detected nitrite by a commercial kit to indirectly reflect NO levels. It should be pointed out that nitrite is both a NO metabolite and a reserve for NO synthesis [34]. Therefore, it is hard to explain whether the increased nitrite by HINT2 overexpression could be interpreted as enhanced NO synthesis. However, increased nitrite has been reported to improve endothelial dysfunction and attenuate infarct size in myocardial IR injury [14], still supporting a protective role of HINT2 in cardiac microcirculation. Under normoxia, without affecting MCU protein levels, the expression of MICU1 and MICU2, as well as [Ca^2+^]_m_ level, was increased after HINT2 overexpression. Whether MICU1 and MICU2 could be directly bound by HINT2 and thereby modulate [Ca^2+^]_m_ influx was not identified. Future studies are warranted to test this hypothesis.

In conclusion, the present study determined that HINT2 acted as a protective factor in the cardiac microvasculature. HINT2 protected cardiac microvascular structure and function against I/R injury and maintained mitochondrial dynamics and function by modulating [Ca^2+^]_m_ through the MCU complex in CMECs. These findings elucidate a new role of HINT2 in cardioprotection and suggest that the MCU complex/[Ca^2+^]_m_ overload/mitochondrial fission/apoptosis signaling pathway is potentially one of the pathological mechanisms underlying microvascular I/R injury.

## Supplementary Information

Below is the link to the electronic supplementary material.

Supplementary file1 AAV9 transfection efficiency in the cardiac microcirculation was detected by immunofluorescence staining of Flag (green), CD31 (purple, pseudo-color) and cTnT (red) in AAV9-NC- or AAV9-HINT2-infected heart. Scale bars: 40 μm (a). Immunofluorescence staining of Flag (green) and HINT2 (red) in AAV9-NC- or AAV9-HINT2-infected heart. Scale bars: 40 μm (b). Western blot analysis was used to quantify HINT2 expression in CMECs and cardiomyocytes isolated from AAV9-NC- or AAV9-HINT2-infected heart (c, d). Evans blue was extracted from the left ventricle and quantified to determine cardiac microvascular permeability (e). VE-Cadherin expression and phosphorylation at Tyr^731^ were detected by Western blot analysis. VE: VE-Cadherin (f). Coimmunoprecipitation (Co-IP) was performed to examine VE-Cadherin and β-Catenin interactions in left ventricle (g). * p<0.05, significantly different as indicated. ns: not significant. (PDF 638 KB)

Supplementary file2 After 45 minutes ischemia and 6 hours reperfusion injury, CMECs were isolated from AAV9-NC or AAV9-HINT2-infected heart. Western blot analysis of DRP1, Fis1, MFN1 and MFN2 in isolated CMECs (a, b). (c-f) CMECs were isolated from 6- to 8-week-old WT mice, transfected with ADV-NC or ADV-HINT2, and subjected to OGD/R injury. DRP1 oligomers were detected by native PAGE and standardized to the DRP1 monomer. DRP1-o: DRP1 oligomers. DRP1-m: DRP1 monomer (c). Mdivi-1 (5 μM) was added to the ADV-NC group during OGD/R injury to inhibit mitochondrial fission. FCCP (1 μM) was applied to ADV-HINT2-infected cells during OGD/R injury for 2 hours to reintroduce mitochondrial fission. Mitochondrial morphology was labeled by Mitotracker (red), and mitochondrial length were quantified. Scale bars: 10 μm (d). Cell viability was measured by CCK-8 assay (e). Cell apoptosis was detected by TUNEL staining and quantified. Scale bars: 60 mm (f). * p<0.05, significantly different as indicated. ns: not significant. (PDF 416 KB)

Supplementary file3 CMECs isolated from 6- to 8-week-old WT mice were exposed to OGD/R injury, with or without Ru360 pretreatment (10 μM, 1 hour). Mitochondrial morphology was stained by Mitotracker (red). Scale bars: 10 μm (a). Fis1 expression and DRP1 phosphorylation at Ser616 were detected by Western blot analysis (b). DRP1 oligomers were detected by native PAGE and standardized to the DRP1 monomer. DRP1-o: DRP1 oligomers. DRP1-m: DRP1 monomer (c). Structure-based protein interaction interface analysis between HINT2 and MCU. 3D Cartoon represents the predicted HINT2-MCU complex structure, in where the potential binding amino acids were depicted. Binding free energy (BFE) was calculated by MMGBSA method (d). The predicted amino acid binding sites and related bases in HINT2, and the mutated bases and amino acids are indicated. All four AAs were mutated into Ala in mutant-1. Leu76, Phe78 and Asp80 were mutated into Ala in mutant-2. Trp160 was mutated into Ala in mutant-3 (e). 3D Cartoon and BFE of HINT2-MCU complex structure after amino acids mutation in HINT2. All the mutants had higher BFE with MCU than wild type, and mutant-2 had the highest BFE. Plasmid containing HINT2 mutant-2 was constructed and transfected into CMECs (f). CMECs were transfected with plasmid containing HINT2 wild type or mutant, and stained for Flag (green), MCU (red) and Mitotracker (purple, pseudo-color). Mut-HINT2: HINT2 mutant. NC: negative control. Scale Bar: 20 μm (g). Co-IP analysis of MCU and Flag-tagged HINT2 mutant in CMECs (h). CMECs were transfected with plasmid containing HINT2 mutant or negative control, and subjected to OGD/R injury. Mitochondrial morphology was stained by Mitotracker (red). Scale bars: 10 μm (i). * p<0.05, significantly different as indicated. ns: not significant. (PDF 727 KB)

Supplementary file4 CMECs were isolated from 6- to 8-week-old WT mice, transfected with ADV-NC or ADV-HINT2, and subjected to OGD/R injury. Cells transfected with ADV-NC were pretreated with Ru360 (10 μM) 1 hour before OGD/R injury to inhibit the MCU complex. Spermine (10 μM) was applied to ADV-HINT2-infected cells during OGD/R injury for 2 hours to reactivate the MCU complex. CMEC migration ability was measured by scratch Wound healing assay (a) and Transwell assay (b). Scale bars: 200 μm. Angiogenesis in vitro was assessed by tube formation assay. BF: Bright field. Scale bars: 300 μm (c). VEGF and VEGFR2 expression were detected by Western blot analysis (d). The interaction between VE-Cadherin and β-Catenin was detected by Co-IP assay. VE: VE-Cadherin (e). * p<0.05, significantly different as indicated. ns: not significant. (PDF 879 KB)

Supplementary file5 Mice were transfected with AAV9-NC or AAV9-HINT2 and suffered from 45 minutes ischemia and 6 hours reperfusion injury. Ru360 (50 nmol/kg) was injected intraperitoneally into AAV9-NC mice 1 hour before I/R injury to inhibit the MCU complex. Spermine (5 mg/kg) was injected intraperitoneally into AAV9-HINT2 mice 1 hour before I/R injury to activate the MCU complex. VE-Cadherin expression and phosphorylation at Tyr^731^ were detected by Western blot analysis. VE: VE-Cadherin (a). Co-IP was performed to examine the VE-Cadherin and β-Catenin interaction (b). Evans blue was extracted from the left ventricle and quantified to determine cardiac microvascular permeability (c). Western blot analysis of ICAM-1 and VCAM-1 (d). * P < 0.05, significantly different as indicated. (PDF 203 KB)

Supplementary file6 Table S1 Primary and secondary antibodies used in immunofluorescent staining. (DOCX 18 KB)

Supplementary file7 Table S2 Primary antibodies used in western blots. (DOCX 21 KB)

## Data Availability

The datasets used and analyzed supporting the findings of this study are available in this paper or the Supplementary Materials. Any other raw data generated or analyzed in this study can be obtained upon reasonable request from the corresponding authors.
