# Supplementary figures and images for: Protective effect of HINT2 on mitochondrial function via repressing MCU complex activation attenuates cardiac microvascular ischemia–reperfusion injury
Source: Basic Res Cardiol. 2021 Dec 16;116(1):65. doi: 10.1007/s00395-021-00905-4 (PMC8677646; doi:10.1007/s00395-021-00905-4)

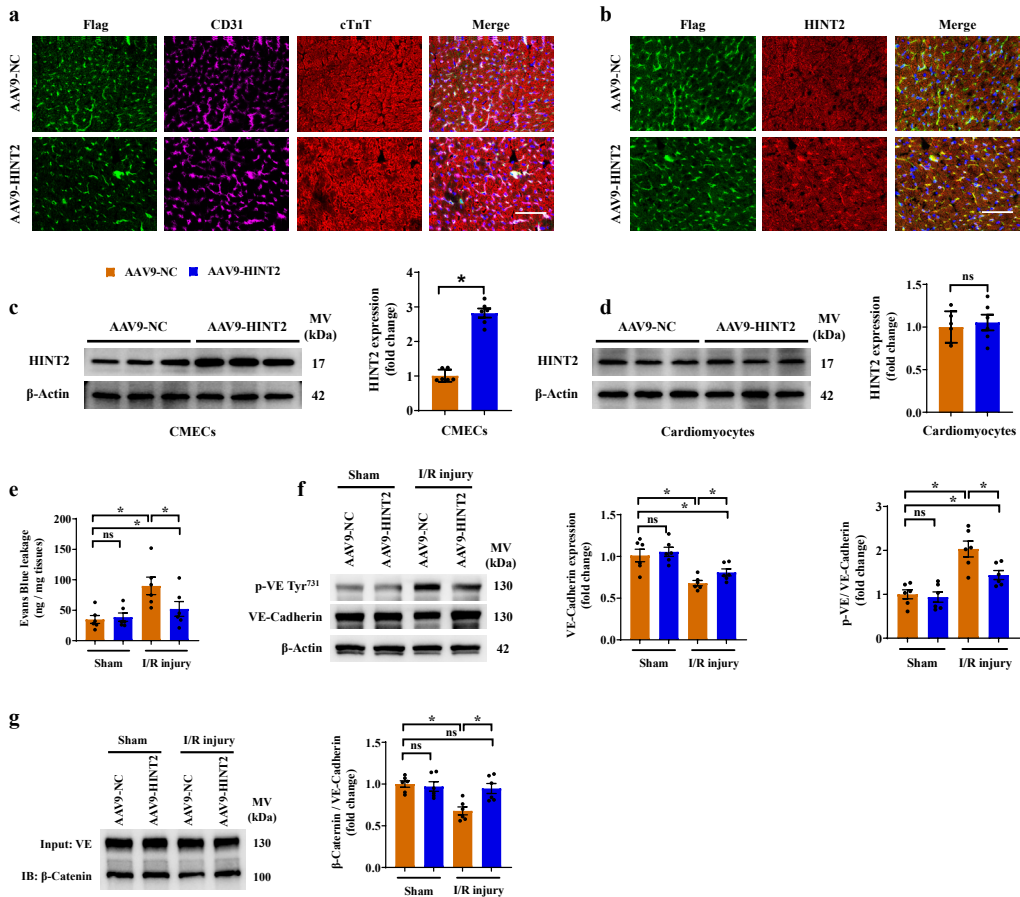

Supplement: Supplementary file 1 — Supplementary file1 AAV9 transfection efficiency in the cardiac microcirculation was detected by immunofluorescence staining of Flag (green), CD31 (purple, pseudo-color) and cTnT (red) in AAV9-NC- or AAV9-HINT2-infected heart. Scale bars: 40 μm (a). Immunofluorescence staining of Flag (green) and HINT2 (red) in AAV9-NC- or AAV9-HINT2-infected heart. Scale bars: 40 μm (b). Western blot analysis was used to quantify HINT2 expression in CMECs and cardiomyocytes isolated from AAV9-NC- or AAV9-HINT2-infected heart (c, d). Evans blue was extracted from the left ventricle and quantified to determine cardiac microvascular permeability (e). VE-Cadherin expression and phosphorylation at Tyr731 were detected by Western blot analysis. VE: VE-Cadherin (f). Coimmunoprecipitation (Co-IP) was performed to examine VE-Cadherin and β-Catenin interactions in left ventricle (g). * p<0.05, significantly different as indicated. ns: not significant. (PDF 638 KB) [file 395_2021_905_MOESM1_ESM.pdf]

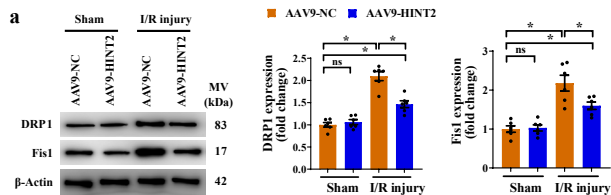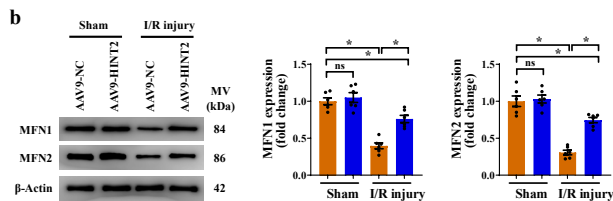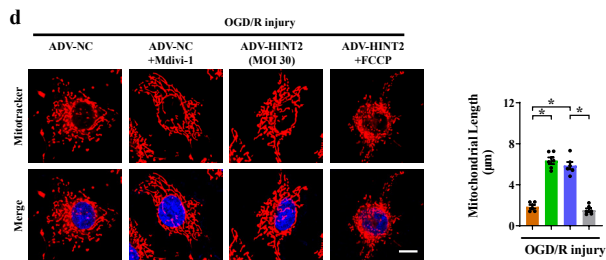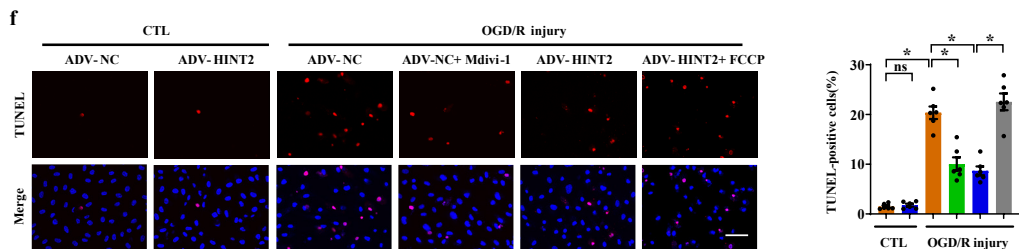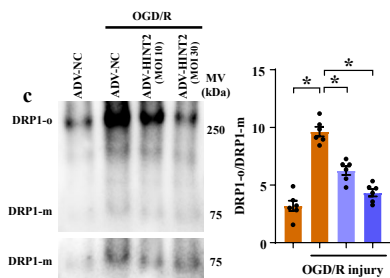

Supplement: Supplementary file 2 — Supplementary file2 After 45 minutes ischemia and 6 hours reperfusion injury, CMECs were isolated from AAV9-NC or AAV9-HINT2-infected heart. Western blot analysis of DRP1, Fis1, MFN1 and MFN2 in isolated CMECs (a, b). (c-f) CMECs were isolated from 6- to 8-week-old WT mice, transfected with ADV-NC or ADV-HINT2, and subjected to OGD/R injury. DRP1 oligomers were detected by native PAGE and standardized to the DRP1 monomer. DRP1-o: DRP1 oligomers. DRP1-m: DRP1 monomer (c). Mdivi-1 (5 μM) was added to the ADV-NC group during OGD/R injury to inhibit mitochondrial fission. FCCP (1 μM) was applied to ADV-HINT2-infected cells during OGD/R injury for 2 hours to reintroduce mitochondrial fission. Mitochondrial morphology was labeled by Mitotracker (red), and mitochondrial length were quantified. Scale bars: 10 μm (d). Cell viability was measured by CCK-8 assay (e). Cell apoptosis was detected by TUNEL staining and quantified. Scale bars: 60 mm (f). * p<0.05, significantly different as indicated. ns: not significant. (PDF 416 KB) [file 395_2021_905_MOESM2_ESM.pdf]

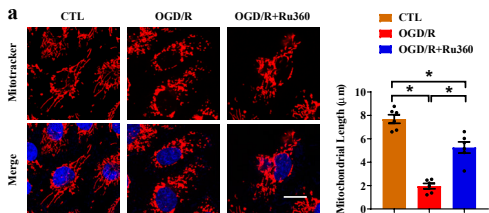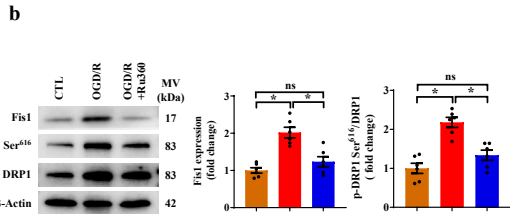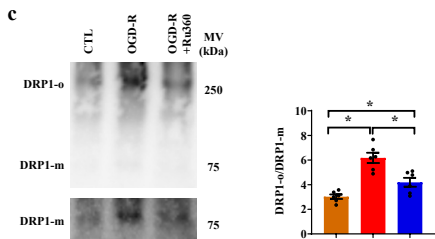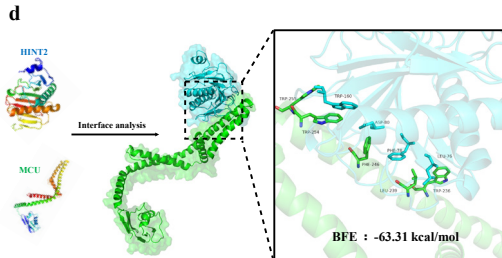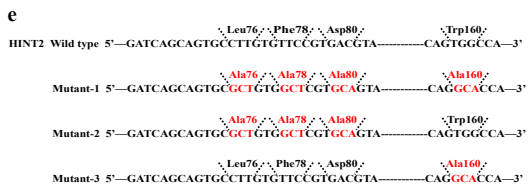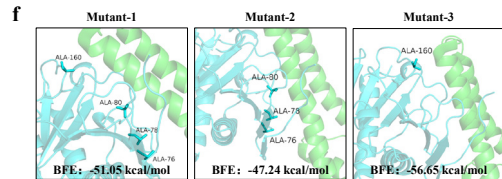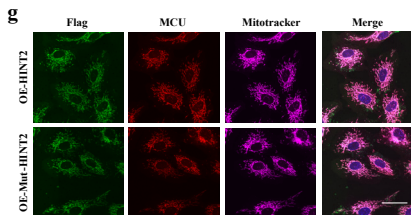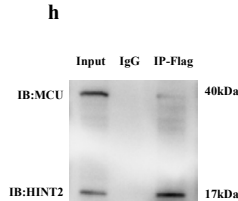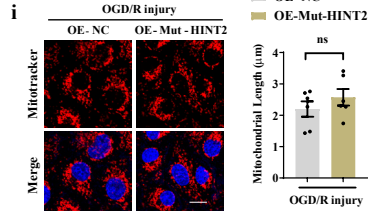

Supplement: Supplementary file 3 — Supplementary file3 CMECs isolated from 6- to 8-week-old WT mice were exposed to OGD/R injury, with or without Ru360 pretreatment (10 μM, 1 hour). Mitochondrial morphology was stained by Mitotracker (red). Scale bars: 10 μm (a). Fis1 expression and DRP1 phosphorylation at Ser616 were detected by Western blot analysis (b). DRP1 oligomers were detected by native PAGE and standardized to the DRP1 monomer. DRP1-o: DRP1 oligomers. DRP1-m: DRP1 monomer (c). Structure-based protein interaction interface analysis between HINT2 and MCU. 3D Cartoon represents the predicted HINT2-MCU complex structure, in where the potential binding amino acids were depicted. Binding free energy (BFE) was calculated by MMGBSA method (d). The predicted amino acid binding sites and related bases in HINT2, and the mutated bases and amino acids are indicated. All four AAs were mutated into Ala in mutant-1. Leu76, Phe78 and Asp80 were mutated into Ala in mutant-2. Trp160 was mutated into Ala in mutant-3 (e). 3D Cartoon and BFE of HINT2-MCU complex structure after amino acids mutation in HINT2. All the mutants had higher BFE with MCU than wild type, and mutant-2 had the highest BFE. Plasmid containing HINT2 mutant-2 was constructed and transfected into CMECs (f). CMECs were transfected with plasmid containing HINT2 wild type or mutant, and stained for Flag (green), MCU (red) and Mitotracker (purple, pseudo-color). Mut-HINT2: HINT2 mutant. NC: negative control. Scale Bar: 20 μm (g). Co-IP analysis of MCU and Flag-tagged HINT2 mutant in CMECs (h). CMECs were transfected with plasmid containing HINT2 mutant or negative control, and subjected to OGD/R injury. Mitochondrial morphology was stained by Mitotracker (red). Scale bars: 10 μm (i). * p<0.05, significantly different as indicated. ns: not significant. (PDF 727 KB) [file 395_2021_905_MOESM3_ESM.pdf]

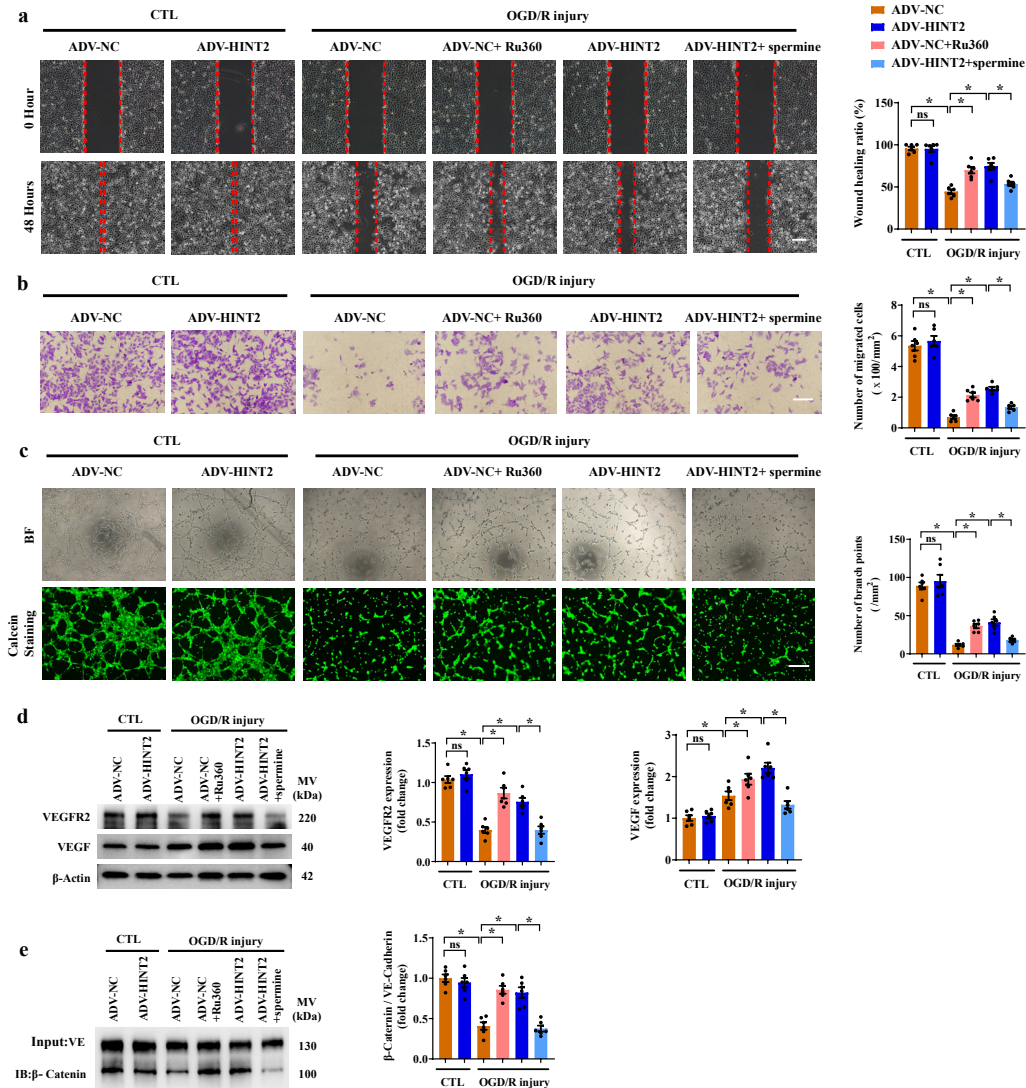

Supplement: Supplementary file 4 — Supplementary file4 CMECs were isolated from 6- to 8-week-old WT mice, transfected with ADV-NC or ADV-HINT2, and subjected to OGD/R injury. Cells transfected with ADV-NC were pretreated with Ru360 (10 μM) 1 hour before OGD/R injury to inhibit the MCU complex. Spermine (10 μM) was applied to ADV-HINT2-infected cells during OGD/R injury for 2 hours to reactivate the MCU complex. CMEC migration ability was measured by scratch Wound healing assay (a) and Transwell assay (b). Scale bars: 200 μm. Angiogenesis in vitro was assessed by tube formation assay. BF: Bright field. Scale bars: 300 μm (c). VEGF and VEGFR2 expression were detected by Western blot analysis (d). The interaction between VE-Cadherin and β-Catenin was detected by Co-IP assay. VE: VE-Cadherin (e). * p<0.05, significantly different as indicated. ns: not significant. (PDF 879 KB) [file 395_2021_905_MOESM4_ESM.pdf]

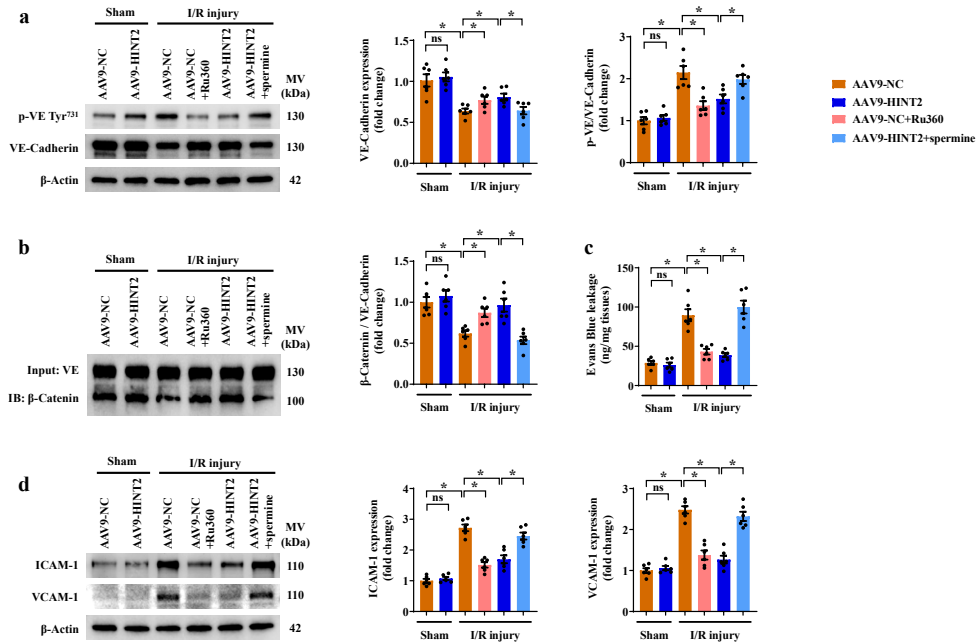

Supplement: Supplementary file 5 — Supplementary file5 Mice were transfected with AAV9-NC or AAV9-HINT2 and suffered from 45 minutes ischemia and 6 hours reperfusion injury. Ru360 (50 nmol/kg) was injected intraperitoneally into AAV9-NC mice 1 hour before I/R injury to inhibit the MCU complex. Spermine (5 mg/kg) was injected intraperitoneally into AAV9-HINT2 mice 1 hour before I/R injury to activate the MCU complex. VE-Cadherin expression and phosphorylation at Tyr731 were detected by Western blot analysis. VE: VE-Cadherin (a). Co-IP was performed to examine the VE-Cadherin and β-Catenin interaction (b). Evans blue was extracted from the left ventricle and quantified to determine cardiac microvascular permeability (c). Western blot analysis of ICAM-1 and VCAM-1 (d). * P < 0.05, significantly different as indicated. (PDF 203 KB) [file 395_2021_905_MOESM5_ESM.pdf]
