# Supplementary material for: Protective effect of HINT2 on mitochondrial function via repressing MCU complex activation attenuates cardiac microvascular ischemia–reperfusion injury
Source: Basic Res Cardiol. 2021 Dec 16;116(1):65. doi: 10.1007/s00395-021-00905-4 (PMC8677646; doi:10.1007/s00395-021-00905-4)
Supplement: Supplementary file 6 — Supplementary file6 Table S1 Primary and secondary antibodies used in immunofluorescent staining. (DOCX 18 KB) [file 395_2021_905_MOESM6_ESM.docx]

Table S1 Primary and secondary antibodies used in immunofluorescent staining.

| Name | Manufacturer | Cat No. | Host | Dilution | Application |
| --- | --- | --- | --- | --- | --- |
| CD31 | Abcam | ab7388 | Rat | 1:500 | IHC-Fr, ICC/IF |
| cTnT | Protein Tech | 15513-1-AP | Rabbit | 1:500 | IHC-Fr |
| Flag | Thermo Fisher | MA1-91878 | Mouse | 1:100 | IHC-Fr, ICC/IF |
| HINT2 | abbexa | abx129872 | Rabbit | 1:200 | IHC-Fr, ICC/IF |
| Albumen | Abcam | ab207327 | Rabbit | 1:200 | IHC-Fr |
| Cytochrome C | Abcam | ab110325 | Mouse | 1:500 | ICC/IF |
| Tomm20 | Abcam | ab186735 | Rabbit | 1:250 | ICC/IF |
| MCU | Novus | NBP2-92310 | Rabbit | 1:200 | ICC/IF |
| VE-Cadherin | Thermo Fisher | 14-1441-82 | Rat | 1:200 | ICC/IF |
| VCAM-1 | Abcam | ab134047 | Rabbit | 1:250 | ICC/IF |
| Donkey anti-Rat AF488 | Thermo Fisher | A21208 | Donkey | 1:500 | ICC/IF |
| Donkey anti-Rat AF594 | Thermo Fisher | A21209 | Donkey | 1:500 | IHC-Fr, ICC/IF |
| Donkey anti-Rat AF647 | Thermo Fisher | A48272 | Donkey | 1:500 | IHC-Fr |
| Goat Anti-mouse AF488 | Abcam | ab150113 | Goat | 1:500 | IHC-Fr, ICC/IF |
| Donkey anti-Rabbit AF488 | Thermo Fisher | A21206 | Donkey | 1:500 | IHC-Fr, ICC/IF |
| Donkey anti-Rabbit AF594 | Thermo Fisher | A21207 | Donkey | 1:500 | IHC-Fr, ICC/IF |
