# Supplementary material for: Protective effect of HINT2 on mitochondrial function via repressing MCU complex activation attenuates cardiac microvascular ischemia–reperfusion injury
Source: Basic Res Cardiol. 2021 Dec 16;116(1):65. doi: 10.1007/s00395-021-00905-4 (PMC8677646; doi:10.1007/s00395-021-00905-4)
Supplement: Supplementary file 7 — Supplementary file7 Table S2 Primary antibodies used in western blots. (DOCX 21 KB) [file 395_2021_905_MOESM7_ESM.docx]

Table S2 Primary antibodies used in western blots.

| Name | Manufacturer | Cat No. | Dilution | Host | Application |
| --- | --- | --- | --- | --- | --- |
| HINT2 | Thermo Fisher | PA5-50226 | 1:1000 | Rabbit | WB |
| HINT2 | abbexa | abx129872 | 1:500 | Rabbit | WB |
| eNOS | CST | 32027S | 1:1000 | Rabbit | WB |
| p-eNOS Ser^1177^ | Abcam | ab215717 | 1:1000 | Rabbit | WB |
| ET-1 | Abcam | ab2786 | 1:1000 | Mouse | WB |
| ICAM-1 | Affinity | AF6088 | 1:500 | Rabbit | WB |
| VCAM-1 | Abcam | ab134047 | 1:2000 | Rabbit | WB |
| DRP1 | CST | 8570 | 1:1000 | Rabbit | WB |
| DRP1 (phospho S637) | Abcam | ab193216 | 1:1000 | Rabbit | WB |
| phospho-DRP1 (Ser616) | CST | 4494 | 1:1000 | Rabbit | WB |
| Fis1 | Affinity | DF12005 | 1:1000 | Rabbit | WB |
| Mitofusin 1 | Abcam | ab221661 | 1:1000 | Rabbit | WB |
| Mitofusin 2 | Abcam | ab124773 | 1:1000 | Rabbit | WB |
| Mn-SOD | Abcam | ab68155 | 1:1000 | Rabbit | WB |
| cytochrome C | Abcam | ab110325 | 1:1000 | Mouse | WB |
| Bax | Abcam | ab182733 | 1:1000 | Rabbit | WB |
| Bcl-2 | Abcam | ab182858 | 1:2000 | Rabbit | WB |
| Caspase9 | Abcam | ab202068 | 1:2000 | Rabbit | WB |
| cleaved-Caspase3 | CST | 9664 | 1:1000 | Rabbit | WB |
| MCU | Novus | NBP2-92310 | 1:1000 | Rabbit | WB |
| MCU | Abcam | ab219827 | 1:500 | Rabbit | WB |
| MICU1 | Thermo Fisher | PA5-77364 | 1:200 | Rabbit | WB |
| MICU2 | Thermo Fisher | PA5-88410 | 1:1000 | Rabbit | WB |
| MCUb | [MyBioSource](https://www.biocompare.com/104355-MyBioSource-com/) | MBS3223833 | 1:250 | Rabbit | WB |
| MCUb | Novus | NBP3-10846 | 1:500 | Rabbit | WB |
| VEGFR2 | Abcam | ab221679 | 1:1000 | Rabbit | WB |
| VEGF | Abcam | ab214424 | 1:1000 | Rabbit | WB |
| VE-Cadherin | Thermo Fisher | 14-1441-82 | 1:1000 | Rat | WB |
| VE-Cadherin | Abcam | ab205336 | 1:1000 | Rabbit | WB |
| p-VE-Cadherin Tyr731 | Affinity | AF3265 | 1:500 | Rabbit | WB |
| COXIV | Abcam | ab202554 | 1:2000 | Rabbit | WB |
| β-Actin | Weiao | WB0196 | 1:1000 | Goat | WB |
